# Pain Management Techniques and Practice: New Approaches, Modifications of Techniques, and Future Directions

**DOI:** 10.1155/2012/239636

**Published:** 2012-09-16

**Authors:** Andrea Trescot, Hans Hansen, Standiford Helm, Giustino Varrassi, Magdi Iskander

**Affiliations:** ^1^Trescot Pain Fellowship, Wasilla, AK, USA; ^2^Pain Relief Centers, Conover, NC, USA; ^3^Pacific Coast Pain Management Center, Laguna Hills, CA, USA; ^4^Department of Anesthesiology, University of L'Aquila, L'Aquila, Italy; ^5^Anaesthesia and Pain Relief, National Cancer Institute, Cairo University, Cairo, Egypt

The practice of pain medicine has radically changed over the last twenty years, morphing from an almost exclusively anesthesia-based, recovery room, and procedure-oriented part-time practice into a multidisciplinary, multimodality, multispecialty field. These changes have been the consequence and the stimuli for the expansion of new medications and techniques, which have improved the diagnosis and treatment of painful conditions. This issue attempts to highlight some of the advances in anesthesiology and pain, including epidural analgesia, spinal cord stimulation, and trigger point diagnosis and treatment. There is also a case report of a technique utilizing transforaminal blood patches to treat intracranial hypotension, analogous to postdural puncture headaches. Particularly intriguing, given the current controversy regarding the role of opioids in the management of chronic pain, is the report of the lasting developmental delays seen in infant rats exposed to fentanyl. This observation could have a significant impact on the decision to initiate opioids in human infants and children and adds data to the current dilemma. 

Pain medicine is a rapidly growing field, and the innovations described in this issue move the field further into the future. Hopefully, the reader will be encouraged to utilize and expand on these topics in their own practice. 

*Andrea Trescot* *Hans Hansen* *Standiford Helm* *Giustino Varras* *Magdi Iskander*

